# Heartbreak and Loneliness Due to Family Separations and Limited Visiting during COVID-19: A Qualitative Study

**DOI:** 10.3390/ijerph20021633

**Published:** 2023-01-16

**Authors:** Wai-King Tsui, Ka-Huen Yip, Yuk-Chiu Yip

**Affiliations:** 1 School of Health Sciences, Caritas Institute of Higher Education, Hong Kong, China; 2 Li Ka Shing School of Professional and Continuing Education, Hong Kong Metropolitan University, Hong Kong, China

**Keywords:** parents, children, pediatric ward, family-centered care, COVID-19 pandemic

## Abstract

The COVID-19 pandemic has greatly impacted the healthcare system. In the pediatric unit, stress, uncertainty, and many unexpected challenges for many parents were frequently reported. Research has shown that parents had less contact with their children during the pandemic due to hospital restrictions. However, it is unknown how parents perceived their experiences in a pediatric unit. This study aimed to describe the lived experiences of parents who had a child in the pediatric unit during the pandemic. A qualitative descriptive approach was used to investigate parents’ experiences of having children admitted to the pediatric unit during the pandemic in Hong Kong. Eight Chinese parents participated in the interview. Three major themes emerged: (1) parents’ pediatric ward experiences during COVID-19 were emotionally isolating and overwhelming, (2) the family and family-centered care were disrupted, and (3) interactions with pediatric providers intensified or alleviated emotional distress. Integrating the above themes of experiences of emotional distress was the main characteristic of the parents’ experiences during the pandemic. Therefore, policymakers should understand the lived experiences of parents of children diagnosed with COVID-19 and should make prompt decisions to deal with both parental concerns and safety issues.

## 1. Introduction

The highly transmissible Omicron variant of COVID-19 has driven an unprecedented surge of infections globally. Government constraints to resist the COVID-19 pandemic has had a profound effect on parents and their children worldwide. Hong Kong has endured five waves of the COVID-19 pandemic thus far, and pediatric wards have been left in a critical condition, with most pediatric units needing to increase their number of beds available for admission. Hong Kong’s isolation centers, hospitals, and morgues have been overflowing. Parents were not allowed to stay with their children upon their admission to a pediatric unit, which was traumatizing for them. They were fearful for their child’s survival given the COVID-19-associated hospitalization rates, mortality increase in Omicron cases, cases of associated complications, and the 2022 report on deaths in Hong Kong by the Department of Health [1]. Parents could neither protect their children, which they considered their responsibility, nor could they act as expert healthcare providers in the face of the lack of care from overworked nurses [2]. Parents felt powerless; they experienced helplessness and uncertainty regarding the appropriate care for their sick children. During the COVID-19 pandemic, most parents of children admitted to pediatric wards reported high levels of uncertainty, anxiety, stress, and decreased parenting confidence [3]. Both fathers and mothers reported significant stress and the need for reassurance and support [4] due to uncertainty and the fact that many Hong Kong citizens were not yet fully vaccinated. A potentially negative impact on the emergent parent–child relationship has resulted from these preventative and restrictive measures. Mobile phones of many new parents were flooded with mixed information from various online media and health organizations in Hong Kong during the rapidly changing and health-threatening COVID-19 pandemic in 2022. This information was not always consistent and was often contradictory.

The hospitalization of children negatively affects parental mental health and parent–child bonding [5]. Some studies show that when children require hospitalization, the practice of parenthood changes, which worsens parental health and confidence [6]. The family-centered approach supposes that the primary mechanisms for child development consist of communication, intercommunication, engagement in activities, attitude, and manners that comprise a child’s different life contexts. Therefore, many hospitals have adopted a family-centered approach to solve these problems and optimize both children’s and parental mental health, as well as mitigating the potential negative impact on the emergent parent-child relationship that arose from the preventative and restrictive measures for COVID-19. Being flooded with varying, often contradictory, information from various sources has resulted in elevated levels of stress and uncertainty among parents [7]. When a child was admitted to the pediatric ward, this likely resulted in further exacerbation of stress for both the parents and child. Recent studies have shown that due to the restrictive policies regarding visiting their children during the COVID-19 pandemic, parents have reported worry [8]. This policy caused a decrease in bonding, especially regarding infants [9]. Recent studies have also described the lived experiences of parents whose children were infected with and were hospitalized due to COVID-19 and how they coped with the impact of pandemic-related policies on their children’s hospitalization. Therefore, it is imperative to develop a comprehensive and detailed understanding of parents’ experiences, their perspectives, and the needs of families in pediatric wards.

### Objectives

This study aimed to describe the lived experience of parents whose children were admitted to pediatric wards during the COVID-19 pandemic. Furthermore, it explored developmental and family-centered care practices for hospitalized children and their families during the pandemic, as well as the implementation of strategies to engage parents in the care of their children. The research questions of the study are (a) “What are parents’ experiences caring for children admitted to pediatric wards during the COVID-19 pandemic?”; (b) “How do parents perceive children’s concerns during the COVID-19 pandemic?”; and (c) “How do parents adapt their practices to meet the health needs of infants who were admitted to pediatric wards during the COVID-19 pandemic?”.

## 2. Methods

### 2.1. Design

We used a descriptive phenomenology approach to explore the lived experiences of parents of children admitted to pediatric wards during the COVID-19 pandemic. This study aimed to explore how parents’ experiences and understanding of the services provided to their children affected their emotional status. The phenomenology study design is more powerful than other designs to assess parents’ lived experiences and it can adequately explore challenging events faced by parents during the hospital stay of their children [10].

Phenomenology is a form of qualitative research that focuses on the study of an individual’s lived experiences within the world and is uniquely positioned to help health professions education (HPE) scholars learn from the experiences of others. This study adopted the transcendental phenomenology approach. Moran mentioned that the transcendental phenomenology approach should have no assumptions, no philosophical or scientific theory, and no deductive logic procedures [11]. No other empirical science or psychological speculations should inform the phenomenology’s inquiry and it should only focus on one individual’s intuition. The purpose is to explore the inner evidence—consciousness [12]. To understand both the phenomenon and lived experiences, one has to rely on sensory perceptions, such as thought, memory, imagination, or emotion [13].

### 2.2. Participants and Procedure

Participating parents were invited for an interview between February 2022 and March 2022 via online media platform groups in Hong Kong, such as “Mother Kingdom” and “Hong Kong Moms”. Parents who were eligible for participation were those who had a child requiring hospitalization in a pediatric ward between 01 February 2022 and 31 March 2022.

All eight participants completed the interview successfully, reaching data saturation. Purposive sampling was used, the participants were recruited from peer support groups from online platforms via telephone interviews that lasted between 45 and 90 min and were audio-recorded. They were sent an introductory letter describing the study and the voluntary nature of their participation and signed the letter to provide informed consent; all participants could withdraw from the study without any consequences. The authors employed privacy measures in the recruitment process; participants were asked to contact only the first author (W.-K.T.) directly, and only researchers had access to the data of this study. Ethical approval was obtained from the research and ethics committee of a local academic institute (HRE210103). Questions about the parents’ demographics, child’s health, hospital-related characteristics, family, social status, pediatric ward environments, and several validated measures related to parent experience were asked, in addition to five open-ended questions shown in Table 1. The open-ended questions were developed considering the research aim through the literature review [5,6,7,8,9] to ensure appropriateness. These questions were chosen to ensure that the data captured accurately reflected the lived experiences of parents of children hospitalized during the peak of the fifth wave of the COVID-19 pandemic in Hong Kong. Participants did not receive any compensation from the researchers. All responses were confidential and anonymous, and they contained no identifying information.

### 2.3. Data Analysis

In this study, the individual perspectives of the experiences that parents had during the hospitalization of their children for treatment of COVID-19 and their emotional status were explored. In particular, a descriptive approach was taken to provide a detailed examination of the parents’ lived experiences. For an analysis of the participants’ personal experiences and perceptions of their children’s hospitalization, all the research team followed the approach of six overlapping steps followed by Colaizzi (1978) in which the researchers perform an active role in the interpretive process [14]. The Consolidated Criteria for Reporting Qualitative Research (COREQ) checklist was applied [15].

Participants were asked five open-ended questions regarding the impact of the COVID-19 pandemic to gauge their experiences and interactions with healthcare providers. NVivo 11 software (QSR International, Burlington, MA, USA)was used in this study to analyze the data. A descriptive, thematic analysis was conducted to identify shared patterns of parents’ experiences in pediatric wards during the pandemic [16]. The constructivist approach was used to center parents’ experiences while accepting the investigators’ role, both in interpreting and synthesizing those experiences in this description [17]. Because the first author (W.-K.T.) is an experienced nurse with pediatric specialty training and has been working in the pediatric unit for over 15 years, they conducted the interview, while all coding and analysis were conducted by two research team members (K.-H.Y. and Y.-C.Y.).

A descriptive thematic analysis was employed in this study. Open coding, topic, and category codes were developed inductively. Some important topics, such as emotional experience and staff interactions, were included in the analysis. All the initial themes were checked against the overall dataset, considering alternative explanations and outliers. Finally, all research team members named and defined the themes by using analytic memo writing [18].

## 3. Results

There were eight respondents in this study, and they all answered the five open-ended questions (100% response rate). All the respondents lived in Hong Kong. Parental, children, and hospital characteristics are illustrated in Table 2.

The respondents answered the questions in phrases, sentences, or up to a full page. Some parents answered questions very expressively, while others just elaborated on their feelings, and some gave unspecific responses that were unrelated to the topic.

Based on our examination of the data, the research team members developed three broad themes: (1) parents’ pediatric ward experiences during the COVID-19 pandemic were emotionally isolating and overwhelming, (2) the frequency of changing policies disrupted family-centered care, and (3) the interactions with the healthcare providers, such as doctors and nurses in the pediatric ward, either intensified or alleviated the emotional distress felt by parents in Table 3. Exemplar quotes of the three themes are displayed in Box 1.

Box 1 **Themes, subthemes, and exemplary quotes.**

Theme 1: Parents’ experiences during the COVID-19 pandemic were isolating and overwhelming.
*Subtheme: Isolation and disconnection*

*“I was not allowed to visit my kid when he was admitted. I nearly burst out due to the unforeseen prognosis and didn’t have the emotional support.” (Parent A).*

*“I couldn’t have imagined how bad it was. Many families’ members were diagnosed with COVID-19, and we and our families, our relatives, could only keep in touch by phone. Everywhere was shut down.” (Parent B).*

*“Both my husband and I were not allowed to visit our child; we could only wait for caring teams to phone us to talk about the child’s condition and progress.” (Parent C).*

*“We, as parents, could not visit our daughter and we were really upset and could not share the caring at this critical moment. We definitely understood the precaution, but it was so taxing on both me and my husband.” (Parent D).*

*Subtheme: Distress and trauma*

*“My husband and I were frightened by the phone call from the nurse that our son’s condition had suddenly changed, and he needed to be put on the ventilator. Justin is our only child, and we were afraid that we would lose him. At that moment, it was like a knife stabbing my heart. Fortunately, he recovered speedily.” (Parent B).*

*“It was extremely challenging. Our daughter was born prematurely and was only discharged from the hospital for six months. During her stay in intensive care, it was already a traumatic experience. Unfortunately, she was diagnosed with COVID, and this was a painful experience for us and a tough time for her to fight the virus.” (Parent E),*

*Subtheme: Intense emotional expressions*

*“It was scary to stay home and wait.” (Parent D).*

*“We were terrified to receive the call. When the nurse called us, we both prayed it was good news and not bad. Once I heard that my child’s condition showed no improvement, it really hurt me. I burst into tears.” (Parent F).*

*“We could only speak to the doctors or nurses very briefly. Whenever there were opportunities to ask, I would often forget what I wanted to ask, or the information became too overwhelming. When my parents or husband came home and I repeated all of this to them, it was heartbreaking all over again.” (Parent H).*
Theme 2: Disruption to the family and family-centered care 
*Subtheme: Parents’ essential caregiver role*

*“The hospital as an institution made many policies in that critical moment that prevented caregivers (the parents) from visiting and caring for their children.” (Parent G).*

*“Parents should have been allowed to visit their children at that essential moment. That separation was like breaking up the family and made me feel so alone. I wanted to share and take care of my child during this pandemic.” (Parent H).*

*“During the hospitalization, my child was unstable for the first few days. I felt my child’s loneliness. As a family, it was better to let someone stay to take care of my child.” (Parent B).*

*“Due to the policy, my child had to be left alone and cared for by the doctors, nurses, and multidisciplinary personnel. It was quite scary for the children to come face-to-face with unfamiliar people during nursing care.” (Parent E).*

*“We were being forced to be apart from our children, while they were left alone to face numerous doctors, nurses, and different healthcare professionals. This policy resulted in a lot of trauma for the children and parents.” (Parent D).*

*Subtheme: Egregious loss*

*Category: Bonding loss*

*“Both of us were not allowed to visit our child until discharge, and the siblings were not able to meet each other. It became harder for the siblings to understand what was happening.” (Parent B).*

*“I felt suddenly that my bond with my son had changed. He was just three months old when he was admitted to the ward. It was our first child, and my husband and I really missed him. And I often cried.” (Parent C).*

*Category: Experience loss*

*“I wanted to exclusively breastfeed my three-month-old baby, and in the hospital, he needed to change to bottle feeding as I could not breastfeed him. I was worried that he would not adapt to bottle feeding and not eat well.” (Parent C).*

*“We could only wait for the nursing staff to arrange face-time through WhatsApp. I disliked this interaction, and my child did not recognize me.” (Parent F).*

*“I felt disconnected from my daughter. I was not able to visit her because I was diagnosed with COVID-19 too.” (Parent H).*

*Category: Loss of time*

*“We live together, eat together – in fact, when my husband was not diagnosed with COVID-19, we needed to separate from each other to reduce the risk of being infected. But when my child recovered and was discharged from the hospital, we still needed to live separately until we had a negative result.” (Parent H).*

*“We and my family were diagnosed with COVID-19 at different times. I needed my mother to take care of him after his discharge. It took a long time to wait for the whole family to get a negative result in the COVID-19 test.” (Parent C).*
Theme 3: Interactions with pediatric isolation ward providers intensified or alleviated emotional distress.
*Subtheme: Support and validation*

*“We understood the precautionary steps that the isolation ward was taking, but maybe it was too difficult for the parents to follow and we needed their support for both of us caring for our child.” (Parent A).*

*“The doctors and nurses could have been more sympathetic and supportive. Whenever they called us to discuss the child’s condition, they seemed to be in a hurry and we could not ask them for clarity about the child’s condition.” (Parent E).*

*“I felt that no one understood the real pain and trauma I was dealing with. He needed to be put on a ventilator for a few days due to respiratory difficulties. I was struggling emotionally and mentally and did not understand what was happening when I received a call from the nurse that my child’s condition had gotten worse. I also felt that the policies were so strict – they caused psychological or mental harm to parents.” (Parent B).*

*Subtheme: Professionalism and consistency*

*“During the hospitalization, I was afraid that my child would be infected by other diseases. I was worried that the nurses were not sanitizing and washing their hands properly before touching my baby.” (Parent E).*

*“The policies and protocols kept changing in both the communities and the hospital. I felt that there was no consistency for the care providers and that this would affect the care given to my child too.” (Parent G).*

*Subtheme: Alienation and inclusion*

*“I was confused with the staff’s explanations. Every day, different nurses contacted me telephonically and their explanations were not always clear.” (Parent H).*

*“I was bothered by the rotation of the staff who worked in the ward. I was afraid that during the rotation they would neglect the treatment and care for my child.” (Parent D).*

### 3.1. Theme 1: Parents’ Pediatric Ward Experiences during the COVID-19 Pandemic Were Emotionally Isolating and Overwhelming

Nearly all the parents reported some emotional and mental impact when their children were admitted to a pediatric ward during the COVID-19 pandemic. The isolating and overwhelming theme of parents’ experiences was organized into three subthemes: (1) isolation and disconnection, (2) distress and trauma, and (3) intense emotional expressions.

#### 3.1.1. Isolation and Disconnection

One of the mothers mentioned her experience emotionally, showing her disconnection from her child and family.

*“I was in Penny Bay for isolation and my child was admitted to the hospital with no family member to take care of him. My family seemed to be breaking up. It was the worst experience I’ve ever had.”*

(Parent C)

During the COVID-19 pandemic, very strict rules and policies were established to limit the visiting hours and restrict the number of visitors. All the pediatric wards under the Hospital Authority were rearranged to accommodate the overflow of cases. The spaces that were formerly used by parents to engage in family-centered care practices were now closed and rearranged to accommodate new admissions. Sometimes the space was used to admit overflow cases from the adult ward. All the restrictions limited or restricted the parents from visiting their children, thereby causing isolation, loneliness, and separation, described by the mother as a painful emotional experience that indicated the isolation and loneliness of the parents (see Box 1).

#### 3.1.2. Distress and Trauma

Stress, difficulty, and the feeling of being overwhelmed were the primary emotional experiences that the parents reported facing. In the interview, parents expressed sentiments, such as “traumatic experiences”, “frightening” (Parent B), and “like a knife stabbing my heart” (Parent E). Some parents mentioned that the restriction policy and the unpredictable prognosis of their children compounded their stress.

*“COVID made my life more difficult. New restrictions and policies confused me, and I could not visit my child. Also, it made my relationship between my husband and my parents difficult.”*

(Parent C)

#### 3.1.3. Intense Emotional Expressions

Significant emotional upheaval was reported by the parents, such as bursting into tears, becoming scared, and feeling terrified to receive phone calls. Parents reported that they experienced fear, worry, and panic. At times they had even forgotten how to enquire about their children’s status when the doctors or nurses in charge called them to report their progress and condition.

*“I often forgot what I wanted to ask or the information became so overwhelming, and when my parents or husband came home and I repeated all of this to them, it was heartbreaking all over again.”*

(Parent H)

Some parents expressed their worries and sadness due to sudden changes in the conditions of their children who needed ventilation care.

*“It’s already heartbreaking due to family separations and limited visiting which worsen their feeling of uncertainty and loneliness.”*

(Parent B)

*“The hospital as an institution formulated many policies in that critical moment that prevented caregivers (the parents) from visiting and caring for their children. But my child is under appropriate treatment in the hospital.”*

(Parent G)

It is important to consider letting the whole family isolate together to make it less stressful for the parents. During the COVID-19 pandemic, all the Hospital Authority hospitals restricted visitations. Only in some expectational cases, for example, critical patients, were their relatives allowed to visit them and only for a short duration. Some mothers emphasized their painful experiences, reporting that when their children were hospitalized during COVID-19 they felt scared, terrified, and lonely. One of the mothers, whose child was three months old and was exclusively breastfed, had to suddenly discontinue breastfeeding and could not embrace her child. This poignant experience—the separation and disconnection between the mother and the child—violated the family-centered approach to caring for children.

*“I feel like I missed out on a lot of happy moments with my baby during the pandemic. I had to stop breastfeeding. I couldn’t bath my baby or change diapers. My husband and I missed these precious moments.”*

(Parent C)

### 3.2. Theme 2: Disruption of the Family and Family-Centered Care

The second major theme was that hospital policies in pediatric units disrupted family-centered development care and caused disturbances in the relationship between parents and children. During the COVID-19 pandemic, parents expressed their confusion due to the frequent policy changes. Because the parents needed to follow the restrictions imposed, they could only know their children’s condition when the nurse or doctor in charge called them.

Within this family theme, two subthemes were derived. First, the parent’s role is important in family-centered care, and the restriction policies undermined it. Second, parents felt isolated, overwhelmed, angry, and confused due to the newly imposed policies, which they perceived as limiting family-centered care.

#### 3.2.1. Parents’ Essential Caregiver Role

Several participants expressed that the limited visitations of their children inhibited their ability to act as an essential caregiver during the hospital stay.

*“I wanted to share and take care of my child in this pandemic.”*

(Parent H)

Although some parents accepted these policies as a precaution to decrease the infection rate in the hospital and society, they still expressed their willingness and their rights to care for their children during hospitalization.

*“During hospitalization, my child was unstable for the first few days, but now her condition is stable with the supportive effort of the nurses. I felt my child’s loneliness. As a family, it was better to let someone stay to take care of my child.”*

(Parent B)

The new policies had an even greater impact on mothers who wanted to breastfeed their children exclusively or parents who wanted to give direct care. Parents reported that the restrictions hindered their ability to provide skin-to-skin care, such as breastfeeding, or day-to-day care, such as bathing their children.

*“I wanted to exclusively breastfeed my three-month-old baby, and in the hospital, he needed to change to bottle feeding as I could not breastfeed him.”*

Barriers presented due to the restriction policies particularly hampered communication among all healthcare professionals. Parents felt physically and emotionally fragile, especially when both they and their children were diagnosed with COVID-19. They had to be separated from each other for treatment.

#### 3.2.2. Egregious Loss

The restriction policies interfered with the parents’ functional role as the main caregiver to the children. The parents experienced loss in three main ways: (1) bonding loss, (2) experiences loss, and (3) loss of time (see Box 1).

Owing to the restriction policies imposed, the parents lost out on the experience of holding, touching, caring for, and kissing their children. Parents worried that the lack of bonding would impact their children’s development. They were not only concerned about the impact that these restrictions would have on bonding but also that they could not spend quality time with their children during hospitalization, which could potentially hamper or negatively affect the strength of the family unit. Some mothers felt that the restriction policies made their separation painful, including the restriction on visiting hours and the limited day-to-day care of the children.

### 3.3. Theme 3: Interactions with Pediatric Providers Intensified or Alleviated Emotional Distress

Healthcare providers, such as nurses, doctors, or other healthcare professionals, would exacerbate the emotional status of the parents. There were three main subthemes identified within this theme: (1) support and validation, (2) professionalism and consistency, and (3) alienation and inclusion.

#### 3.3.1. Support and Validation

Parents needed great support and sympathy from the healthcare providers during the hospitalization period. The support offered by the staff helped them overcome their emotional distress. Some parents reported that the nurses reassured them by reporting and explaining their child’s condition in detail and sharing the treatment regimen with them over phone calls.

*“The nurses were amazing. They explained to me in detail and let me see my son via WhatsApp. This comforted me a lot but face-to-face caring for my child would still have been better.”*

(Parent D)

#### 3.3.2. Professionalism and Consistency

Some parents reported that the staff’s responses were inconsistent, especially the junior staff and that there was a lack of professionalism in the conversation between the staff and the parents.

*“During the hospitalization, I was afraid that my child would be infected by other diseases. I was worried that the nurses were not sanitizing and washing their hands properly before touching my baby.”*

(Parent E)

*“The policies and protocols kept changing in both the communities and the hospitals. I felt that there was no consistency for the care providers and that this would affect the care given to my child too.”*

(Parent G)

#### 3.3.3. Alienation and Inclusion

The loss of mutual understanding and failure of acceptance arose, which were mainly attributable to the changes in measures to handle COVID-19. This included the exclusion of parents from rounds, remote consults, perceptions of staff shortages, high staff turnover, reduced parent services, social distancing, and masking, among others.

*“I was confused with the staff’s explanations. Every day, different nurses contacted me telephonically and their explanations were not always clear.”*

(Parent H)

Parents focused more on turnover, which compromised infant care and increased opportunities for potential exposure to COVID-19, and the absence of mutual understanding about the healthcare system and the situation of the healthcare providers. Parents considered the difficulties being faced by themselves rather than the difficulties being faced by healthcare providers. Their attention was solely focused on the restrictions that interfered with their right to care for their children, either by being there and taking care of things physically or by giving feedback to the hospital management.

## 4. Discussion

The COVID-19 pandemic presented immense challenges for both healthcare professionals and parents. The uncertainty of this disease posed serious unprecedented challenges to the healthcare system. In response to this pandemic, a rapid response was necessary to maintain the safety of patients, and policies needed to be set up to respond suitably. The newly imposed policies have long-term and short-term effects, which may be significant [19]. 

During the pandemic, parents whose children required hospitalization reported going through painful experiences, such as separation, loneliness, isolation, and disconnection. These descriptions are very similar to those of other studies conducted during the lockdown periods [20,21]. Before the pandemic, most pediatric wards encouraged family-centered care. Parents and relatives were encouraged to visit their children and be involved in their care; families were also offered psychological support. The painful experiences during the lockdown periods are, however, in contrast to the way things were in the pre-COVID-19 era. The parents also complained about the inconsistency of care and frequent turnover of healthcare providers while their children were hospitalized. These issues led to a loss of mutual trust between the parents and the healthcare providers, and the parents’ feelings of abandonment strengthened [22]. During times of uncertainty, parents’ needs should be considered, and psychological and nursing care support should be provided to them accordingly. Good communication through sharing in the decision-making for their child’s care and having a say in policymaking must be encouraged to mitigate the feelings of the loss of control and uncertainty experienced by parents [23]. 

This study revealed that during the pandemic, parents were overwhelmed with the emotional strain caused by the broken relationship with their children. Parents expressed that their feelings of loneliness and uncertainty were dismissed by some healthcare providers. Some of the parents felt frustrated when their uncertainties were left unaddressed and they received unclear explanations from healthcare providers. Healthcare providers need to provide unique support for the unique needs of families. This study indicated that more rooms should be provided to facilitate parental skills in assisting the children’s adaptation to their new environment. Another important aspect is the consistency of care. For example, there should be a nurse in charge who would be responsible for the case until the discharge of the patient. This provides consistency in care as the healthcare provider would gain familiarity with the case, which would enable them to offer appropriate support to both the parents and the child [24]. The parental perceptions of illness are strongly related to the health of the family [25]. This study is even more crucial during times of significant stress, such as that experienced during the COVID-19 pandemic [6]. Consistency in care and support is important to reduce parental stress. Lastly, inviting families’ input during the formulation of new policies is also important because parents can better explain their needs and provide real feedback for setting up decision-making models of care.

### 4.1. Implications

The study provides information for policymakers and healthcare professionals worldwide regarding limited hospital visits during the COVID-19 pandemic. It also demonstrates the value of caregiver hospital visitations in future pandemic planning. From a caregiving perspective, parents whose children were admitted to pediatric wards can be seen as external partners or an important and integral part of a patient’s care team, as suggested by family-centered approach to care for children. So, in time, adequate psychological support should be offered to parents to mitigate their hardship. The formation of a flexible visiting policy would create an environment conducive to meeting the requirements of the parents and children. Therefore, nurses are required to deliver continuous updates and clarifications to parents to lessen the parents’ misconceptions and misgivings regarding the care that their children receive during prolonged hospital stays.

### 4.2. Limitations

This study’s findings were limited to a small sample—a group of parents whose children were diagnosed with COVID-19 and hospitalized; the sample was homogeneous. The study was carried out during the peak of the pandemic. This, coupled with the fact that most of the participants were willing to answer all the questions telephonically, means that data collection was performed virtually due to the COVID-19 pandemic. The selection criteria were thus biased, which may not accurately reflect the real situation and cannot be generalized. Therefore, further research should include a larger sample that would include more parents of children who were hospitalized in different types of hospitals, such as private or government hospitals, and different pediatric units, with differing durations of stay and reasons for admission. This would provide better sampling for the parents’ perceptions of visitation restrictions. 

A larger sample size would allow for data collection from different minorities within the community to make the data more substantial and comprehensive. Additionally, results may be biased by the participants due to their children’s length of stay in the hospital and sudden changes in policies, which would influence their perceptions.

## 5. Conclusions

This qualitative study covered lived experiences of parents whose children were diagnosed with COVID-19 and required isolation care in pediatric wards during the pandemic. It highlighted the experiences of parents during the pandemic; their emotional pain caused by loneliness, lack of support, feelings of isolation, and lack of family-centered care; and an increase in emotional distress. The parents reported feeling frustrated and disappointed with the visitation restrictions. The research findings apply to all healthcare providers, administrators, and policymakers involved in taking care of the health and welfare of children, and, in turn, their families.

This study proposes that policymakers should attempt to understand the lived experiences of parents of children diagnosed with COVID-19 and make prompt decisions to deal with both parental concerns and safety issues. Furthermore, it is recommended that healthcare providers adopt better communication strategies to reduce parents’ distress, such as identifying the knowledge demands of both children and parents, offering supportive services, increasing the comfort of the parents and their children, providing emotional support, and making better use of online facilities for consultation. It also suggests that family-centered care should be encouraged while formulating care policies for children. Additionally, it is suggested that policies be set up to better equip the healthcare system to deal with crises of similar proportions.

## Figures and Tables

**Table 1:** Probing open-ended questions.

No.	Probing Questions
1.	Can you tell me how you communicated with the healthcare providers in the pediatric ward during the COVID-19 pandemic? If applicable, was there anything healthcare providers could have done differently?
2.	How has the COVID-19 pandemic impacted your transition home?
3.	If you have anything more you would like to share with us about your experience or this interview, please share here.
4.	Do you believe your experience of having a child in the pediatric ward has changed because of the COVID-19 pandemic? Please share more about how your experience in childcare has been impacted by the COVID-19 pandemic.
5.	Recalling your experience of visiting the pediatric ward, what are your feelings toward limited visits during the COVID-19 pandemic?

**Table 2:** Parent, children, and hospitalization characteristics.

Parent Characteristics	
Age	33 to 36
Number of individuals at home	2–4 (all are nuclear families)
Total respondents	8
Number of children	
1	4
2	4
Marital status	
Married/living with a partner	8
Education	
College graduate	2
Graduate/Professional degree	6
Income	
HKD < 35,000	2
HKD 35,000–70,000	4
HKD > 75,000	2
Insurance (not included because all confirmed cases need to be admitted to the Hospital Authority isolation ward)	
Length of stay in the isolation ward (days)	
<5 days	4
6–14 days	4
Reason for admission	
Rapid Antigen Detection Test positive with mild symptoms	4
Rapid Antigen Detection Test positive with respiratory distress	4
Age of the children	
1 month to 1 year old	2
2–3 years old	4
4–6 years old	2

**Table 3:** Themes and subthemes.

Themes	Subthemes
Parents’ experiences during the COVID-19 pandemic were isolating and overwhelming.	Isolation and disconnection
	Distress and trauma
	Intense emotional expressions
Disruption to the family and family-centered care	Parents’ essential caregiver role
	Egregious loss
Interactions with pediatric isolation ward providers intensified or alleviated emotional distress	Support and validation
	Professionalism and consistency
	Alienation and inclusion

## Data Availability

The interview data have been provided in the manuscript. To protect participants’ privacy, the transcripts that contain private and confidential data will not be made publicly available.
